# COVID-19-Induced Seizures: A Meta-Analysis of Case Series and Retrospective Cohorts

**DOI:** 10.7759/cureus.28633

**Published:** 2022-08-31

**Authors:** Helai Hussaini, Sylvette Rogers, Saurabh Kataria, Khalid Uddin, Khalid H Mohamed, Alaa S Mohamed, Farhan Tariq, Sarfaraz Ahmad, Anum Awais, Zahoor Ahmed, Anthony Chukwurah, Aadil Khan

**Affiliations:** 1 Neurology, Toronto General Hospital, Toronto, CAN; 2 Neurology, Caribbean Medical University, Atlanta, USA; 3 Neurology, Ochsner Louisiana State University Health Sciences Center, Shreveport, USA; 4 Neurology and Neurocritical Care, University of Missouri Health Care, Columbia, USA; 5 Neurology, West Virginia University, Morgantown, USA; 6 Neurology, Henry Ford Health System, Detroit, USA; 7 Neurology, Sheffield Teaching Hospitals NHS Foundation Trust, Sheffield, GBR; 8 Neurology, Augusta University, Augusta, USA; 9 Internal Medicine, Dow University of Health Sciences, Karachi, PAK; 10 Internal Medicine, Saint James School of Medicine, Chicago, USA; 11 Internal Medicine, Fatima Jinnah Medical University, Lahore, PAK; 12 Internal Medicine, Mayo Hospital, Lahore, PAK; 13 General Medicine, Apex Specialist Hospital, Awka, NGA; 14 Internal Medicine, Lala Lajpat Rai Hospital, Kanpur, IND

**Keywords:** covid-19, coronavirus disease 2019, epilepsy, seizure, neurological manifestations

## Abstract

The adverse events and complications of coronavirus disease 2019 (COVID-19) continue to challenge the medical profession despite the worldwide vaccination against the severe acute respiratory coronavirus 2 (SARS-CoV-2), the causative agent of COVID-19. Other than typical respiratory manifestations, COVID-19 also presents a wide range of neurological manifestations. This article underlines the pooled incidence of COVID-19-induced seizures in patients with epilepsy and without epilepsy. Following Preferred Reporting Items for Systematic Reviews and Meta-Analyses (PRISMA) protocols, we conducted a bibliographical search, and an initial search revealed 1,375 articles. In total, 21 articles were included in the final analysis by following the inclusion criteria. A total of 11,526 patients from 21 published articles that met the predetermined search criteria were included. The median age of the patients was 61.9 years, of whom 51.5% were males. A total of 255 patients presented with seizures as the first manifestation of COVID-19 with a prevalence of 2.2% (95% confidence interval = 0.05-0.24, *p* < 0.01) (*I*^2^ = 97%), of which 71 patients had previously been diagnosed with epilepsy. Among patients with epilepsy, 49 patients had seizures as an initial presentation of SARA-CoV-2 with an incidence of 72% (0.54-0.85, *p *= 0.1) (*I*^2^ = 34). Although the incidence of COVID-19-induced seizures is not high compared to other neurological manifestations, seizure incidence in epileptic patients with COVID-19 is remarkably high. New-onset seizures in any patient should be considered a presentation of COVID-19 in the absence of other causative factors.

## Introduction and background

The adverse events and complications of coronavirus disease 19 (COVID-19) continue to challenge the medical profession despite the worldwide vaccination against the severe acute respiratory coronavirus 2 (SARS-CoV-2), the causative agent of COVID-19. Other than typical respiratory manifestations, COVID-19 also presents with neurological manifestations [1,2]. SARS-CoV-2 seems to have been transmitted from infected bats to humans and can spread through human-to-human transmission [3]. Patients with COVID-19 typically present with respiratory manifestations ranging from a mild cough to lung infection and respiratory failure in severe cases and can involve other body systems, including gastrointestinal, renal, and cardiovascular systems [4-9]. Many clinical trials for potential therapy and vaccines have combated this pandemic [10-14].

There is also growing evidence that COVID-19 can affect the nervous system, leading to several neurological manifestations and adverse events. Due to its neurotropic and neuroinvasive potential, the data on neurological involvement in COVID-19 has mounted rapidly with an exponential increase in publications [15,16]. Neurological manifestations of COVID-19 include headache, encephalopathy, myalgias, and dizziness, with more severe symptoms including anosmia, peripheral neuropathy, ataxia, seizure, acute cerebrovascular disease, and myopathies [17,18].

Patients with neurological involvement in the setting of COVID-19 infection are at risk of developing seizures due to hypoxia, metabolic derangements, intoxication, and organ failure. Seizures precipitated by COVID-19 may affect the functional outcomes in critically ill patients. This article summarizes the evidence of seizure occurrences in COVID-19 patients and the prevalence of seizures in patients with epilepsy diagnosed with COVID-19.

## Review

Methodology

**Figure 1:**
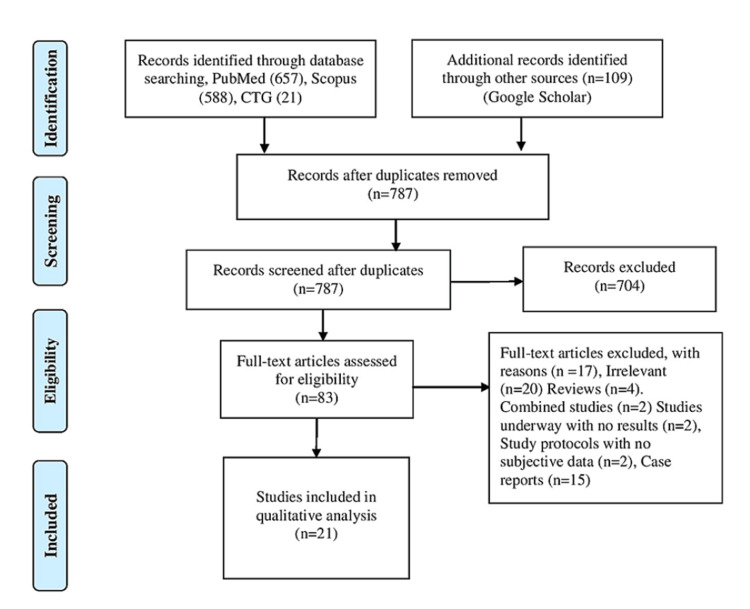
PRISMA flow diagram. PRISMA: Preferred Reporting Items for Systematic Reviews and Meta-analyses; CTG: clinicaltrials.gov

We performed this systematic review and meta-analysis by following the Preferred Reporting Items for Systematic Reviews and Meta-Analyses (PRISMA) guidelines (https://prisma-statement.org//) (Figure 1).

Study Selection and Eligibility Criteria

An extensive bibliographical search was conducted on PubMed and Google Scholar. The initial screening identified 1,375 articles using mesh terms and keywords for COVID-19, seizure, and epilepsy. The two authors screened the articles using predetermined screening criteria, retrieved the relevant articles in full text, and further screened them based on eligibility criteria. Case reports, articles published before 2020, and articles not addressing COVID-19-associated neurological aspects, particularly seizures, were excluded. Case-control studies, case series, and retrospective and prospective cohorts highlighting data on COVID-19 and seizures related to incidence and prevalence were included. Two authors assessed the relevant articles and resolved the disagreements through systemic discussion.

Data Extraction and Statistical Analysis

Two authors extracted the relevant and appropriate data using a Microsoft Excel standard extraction sheet. The relevant data included a proportion of infected patients with seizures, with control data for preexisting epilepsy and alternate provoking causes. Additional retrieved data included author(s), study design, gender, median age, comorbidities, and seizure as an initial manifestation of COVID-19. The quality of included studies was assessed through the Newcastle-Ottawa Quality Assessment Scale (NOS). Any conflicts were resolved through consensus. The publication bias was evaluated using a random-effect funnel plot model.

We performed a random effect analysis to determine the pooled incidence of COVID-19-induced seizures and 95% confidence intervals (CI) using the R programming language (v 4.0.2) [19]. We also estimated the seizure incidence in patients with epilepsy diagnosed with COVID-19. The study heterogeneity was assessed by the *I*^2^ test, which estimates the proportion of total variation among included literature. In case of high heterogeneity, a subgroup analysis was performed based on the location of the studies.

Results

Our study included 21 studies involving nine case series and 12 retrospective cohorts. Included studies had reported seizures as an initial manifestation of COVID-19. COVID-19 confirmation testing was performed through nasopharyngeal or oropharyngeal swabs using real-time polymerase chain reaction (PCR) in all studies. Data on author(s), publication year, number of infected SARS-CoV-2 patients, number of patients presenting with seizure as an initial COVID-19 presentation, and number of patients with epilepsy are highlighted in Table 1 [4,5,17-35].

**Table 1:** Characteristics of included studies. NR: not reported; ICU: intensive care unit; COVID-19: coronavirus disease 19; NA: not available

Author	Study type	Location	Median age (years)	Male (%)	Patient characteristics	COVID-19 patients	Patients with seizure	Patients with epilepsy	Neurological comorbidity
Anand et al. [20]	Case series	United States	75	29	All hospitalized	7	7	3	Stroke 1, Parkinson’s disease 1
Canham et al. [21]	Case series	United Kingdom	55.9	80	ICU patients	10	6	1	Stroke 1
Chen et al. [22]	Case series	United States	45	40	ICU patients	5	3	NR	NR
Delorme et al. [23]	Case series	France	66.8	50	All hospitalized	4	1	1	NR
Galanopoulou et al. [24]	Case-control study	United States	63.2	63.6	ICU patients	28	14	4	Neurological disorders 7
Garazzino et al. [4]	Retrospective, multicenter	Italy	2.3	55.9	Pediatric patients	168	5	4	NR
Jain et al. [25]	Retrospective, multicenter	United States	66	NA	All hospitalized	3218	68	NR	Stroke 35, Encephalitis 1
Louis et al. [26]	Case series	United States	66.5	63.6	All hospitalized	22	5	2	Stroke 1, headache 1
Mao et al. [18]	Retrospective, observational	China	52.7	40.7	All hospitalized	214	2	NR	Cerebrovascular disease 15
Mahammedi et al. [27]	Multicenter, retrospective,	Italy	NA	NA	All hospitalized	725	10	NR	Stroke 34
Petrescu et al. [28]	Case series	France	63.9	80.5	All hospitalized	18	2	1	Stroke 3, brain tumor 1, subdural hematoma 2
Pellinen et al. [29]	Case series	United States	64	71.2	ICU patients	111	42	13	Stroke 13
Pilato et al. [30]	Case series	United States	63	62.5	All hospitalized	8	2	5	Dementia 1, developmental delay 2
Pinna et al [15]	Multicenter, retrospective	United States	59.6	58	ICU	50	13	NR	NR
Radmard et al. [17]	Case series	United States	56.1	20	All hospitalized	33	9	5	NR
Romero-Sánchez et al. [31]	Multicenter, retrospective	Spain	66.42	56.2	All hospitalized	841	6	21	Stroke 53, cognitive impairment 71
Kremer et al [32]	Retrospective, single center	France	61	81	Critical patients	37	5	1	Stroke 7, other neurological disorders 8
Tomlins et al. [33]	Retrospective, single center	United Kingdom	75	63	All hospitalized	95	1	NR	Neurological disease 14
Li et al. [34]	Retrospective, single center	China	3	18	All hospitalized	22	5	NR	NR
Keshavarzi et al. [35]	Retrospective, single center	Iran	58	42	All hospitalized	5872	45	4	NR
Santos de Lima et al. [36]	Retrospective, single center	United States	61.9	56.25	All hospitalized	38	4	6	NR
Total = 21			Age range = 3–75	Male (range) = 80–18		11,526	255	71	

**Figure 2:**
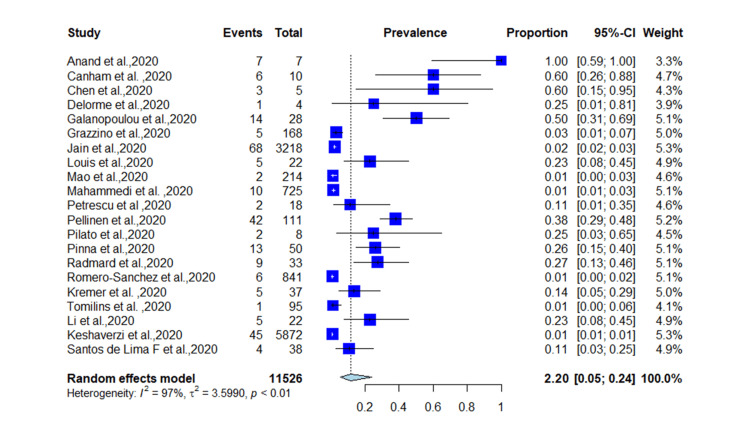
Prevalence of seizures in COVID-19 patients. COVID-19: coronavirus disease 2019 [4,5,17-35].

**Figure 3:**
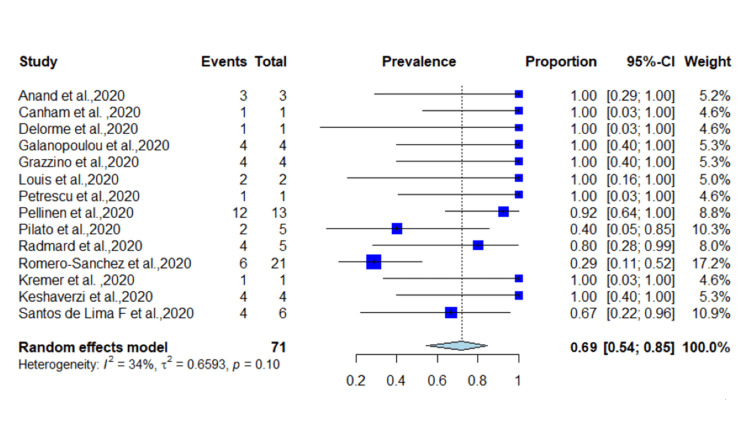
Incidence of seizures in patients with epilepsy having COVID-19. COVID-19: coronavirus disease 2019 [4,17,20,21,23,24,26,28-32,35,36].

A total of 11,526 patients from different countries were identified, with a median age of 61.9 years; 51.5% of the patients were male. In total, 255 patients presented with seizure as the first manifestation of COVID-19 with a prevalence of 2.2% (95% CI = 0.05-0.24, *p* < 0.01) (*I*^2^ = 97%) (Figure 2). Fourteen studies reported epilepsy as neurological comorbidity, and 71 patients were diagnosed with epilepsy before COVID-19 infection with a proportion of 0.98% (0.03-0.018, *p* < 0.01). Among patients with epilepsy, 49 had seizures as an initial presentation of SARS-CoV-2 with an incidence of 69% (0.54-0.85, *p* = 0.1) (*I*^2^ = 34) (Figure 3). The random-effect funnel model shows an association between COVID-19 and seizure occurrence with publication bias.

**Figure 4:**
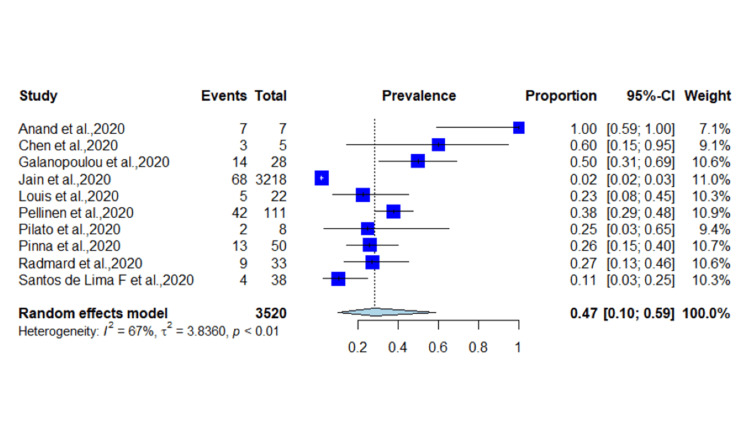
Incidence of seizures in studies from the United States. COVID-19: coronavirus disease 2019 [15,17,20,22,24-26,29,30,36].

**Figure 5:**
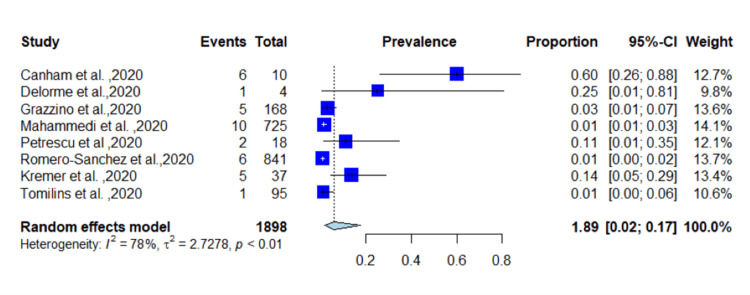
Incidence of COVID-19-induced seizures from European studies. COVID-19: coronavirus disease 2019 [4,21,23,27,28,31-33].

**Figure 6:**
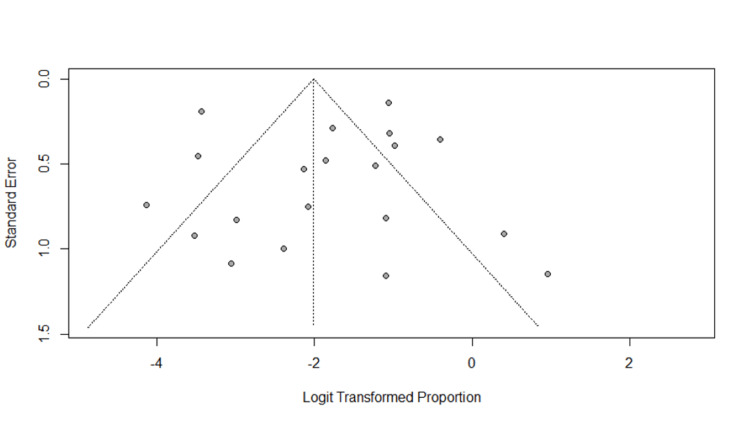
Publication bias in included studies.

Due to high heterogeneity, we performed a subgroup analysis based on the location of the studies. We performed a pooled analysis of American and European studies. In total, 12 studies were from the United States and included 3,520 patients diagnosed with COVID-19. A total of 167 patients had COVID-19-induced seizure with a pooled prevalence of 4.7% (95% CI = 0.10-0.59, *p* < 0.01) (*I*^2^ = 67%) (Figure 4). Eight studies were from Europe and included 1,898 patients diagnosed with SARS-CoV-2. A total of 36 patients had COVID-19-induced seizure with an incidence of 1.89% (95% CI = 0.02-0.17, *p* < 0.01) (*I*^2^ = 78%) (Figure 5). Publication bias in included studies is shown in Figure 6.

Discussion

The adverse events and complications of COVID-19 continue to challenge the medical profession despite the worldwide vaccination against COVID-19. There are reports of cerebrovascular adverse events associated with COVID-19 infection. Acute symptomatic seizure is one of the least reported neurological presentations in COVID-19 patients. Once the pandemic had gained momentum, the number of reports of seizure occurrence in COVID-19 patients increased. Our study highlighted the proportion of patients with preexisting epilepsy who experienced seizure exacerbation as a manifestation of COVID-19 and the proportion of patients who experienced provoked seizures due to COVID, which has significant implications for further management. Although the incidence of COVID-19-provoked seizures is not high, the incidence of seizures in COVID-19 among epileptic patients is high.

Recently, there has been an increase in the number of cases of seizures in patients with COVID-19 infection [15]. The growing literature has documented the neurotropic properties of COVID-19 due to angiotensin-converting enzyme-2 (ACE2) receptors in the nervous system [24]. Seizures were also highlighted in the preceding epidemics of coronavirus infections during the SARS coronavirus infections in 2002 and the Middle East Respiratory Syndrome (MERS) coronavirus infections in 2012, with proportions of 1.9% and 8%, respectively [37,38]. The current pandemic has dramatically affected the population, and several patients have presented with seizures as an initial or the earliest manifestation of COVID-19 [31]. The pathophysiology behind the occurrence of seizures is not yet understood; however, some hypotheses can be postulated. ACE2 receptors for viral entry into the nervous system are predominantly present in the brainstem [3]. After the invasion, SARS-CoV-2 triggers a cascade of reactions leading to the production of inflammatory and proinflammatory cytokines, which result in neuronal hyperexcitability and seizures. Proinflammatory cytokines induce glutamate release and inhibit the release of inhibitory neurotransmitters in the hippocampus and cerebral cortex, leading to seizures and epilepsy [27]. COVID-19 can disrupt the respiratory and cardiovascular systems leading to hypoxia, and hypoxia-induced cerebral damage induces a higher neural activity [39]. Other mechanisms included disruption of the blood-brain barrier, multiorgan failure, severe metabolic derangements, electrolyte abnormalities, and coagulation cascade activation through the production and excessive release of proinflammatory cytokines [40].

Prior smaller studies have highlighted the incidence and prevalence of seizures in patients diagnosed with COVID-19. A retrospective study from the United States reported that the prevalence of COVID-19-induced seizures was 2.1% among 3,218 patients [25]. Another study reported that 26% of patients with seizures were admitted to the hospital as a COVID-19 presentation among 50 infected patients [15].

Favas et al. performed a pooled analysis of seizure incidence in COVID-19 patients. This study included 2,043 patients from five studies and reported a prevalence of COVID-19-induced seizures of 1.1% (CI = 0.7-1.7%) [41]. Another analysis on COVID-19-induced seizures included 314 infected patients and reported a 0.5% incidence (95% CI = 0.02-6.04, *p* = 0.08), and a 0.3% incidence of status epilepticus (95% CI = 0.00-3.69) [40]. COVID-19 incidence in patients with epilepsy is not widely described in the literature, and limited data are available on the prevalence of COVID-19 infection in epilepsy. Garcia et al. reported that COVID-19 incidence in epileptic patients was 1.2% compared to the normal population (0.6%) [42]. An increase in seizure exacerbation in patients diagnosed with epilepsy has also been reported during the pandemic [40,42]. Similar results were observed in our analysis. An interesting observation from our data is that the prevalence of seizures in COVID-19 patients with epilepsy is high.

Seizure exacerbation in patients with epilepsy is linked with prior history of COVID-19 during a pandemic. Multiple stress factors during the pandemic, undesirable outcomes of the infection on seizure-associated health conditions, or noncompliance/change in antiepileptic drugs had also led to seizure exacerbation in epileptic patients. A recent article highlighted that 30.3% of epileptic patients with a history of COVID-19 infection experienced increased seizure exacerbations, and only 7% of patients with epilepsy without exposure to COVID-19 underwent increased seizure exacerbation [43].

Our study has many limitations. Our research has high heterogeneity because many studies in our analysis have a small sample size and moderate quality. We also included case series in our research. We may have a remarkable publication bias in both pooled prevalence likely due to small case series and a less likely chance of negative study publication. Observational studies may have residual confounding. We could not find individual data in a few studies; therefore, we could not make our adjustments, leading to potentially incomplete data. In some publications, the number of patients with epilepsy was not reported. Finally, increasing published data makes retrieving relevant data on the topic difficult.

Our study also highlighted an increased prevalence of COVID-19-induced seizures raising many queries. It can be due to different virus strains, more studies reported from Europe, potentially biased studies with small sample sizes, or different physiological/emotional responses to the pandemic, which are needed to explain in future studies from the rapidly growing data.

## Conclusions

Although seizure prevalence in COVID-19-infected patients is not high compared with other neurological manifestations, new-onset seizures in any patient can raise suspicion of a presentation or complication of COVID-19 infection in the absence of other causative factors during this pandemic. People with epilepsy diagnosed with COVID-19 infection reported increased seizures during the pandemic. Therefore, a comprehensive clinical picture and neurological investigations, including imaging modalities, are mandated while examining and managing such patients. Data from large cohorts are required to better understand this apparent association between seizures and COVID-19 infection, its etiology, increase in seizures in epileptic patients, prognosis, and follow-up protocols for these patients.

## References

[1] Jiang F, Deng L, Zhang L, Cai Y, Cheung CW, Xia Z (2020). Review of the clinical characteristics of coronavirus disease 2019 (COVID-19). J Gen Intern Med.

[2] Singh R, Kashyap R, Hutton A, Sharma M, Surani S (2020). A review of cardiac complications in coronavirus disease 2019. Cureus.

[3] Zhou P, Yang XL, Wang XG (2020). A pneumonia outbreak associated with a new coronavirus of probable bat origin. Nature.

[4] Garazzino S, Montagnani C, Donà D (2020). Multicentre Italian study of SARS-CoV-2 infection in children and adolescents, preliminary data as at 10 April 2020. Euro Surveill.

[5] Menon T, Sharma R, Kataria S (2021). The association of acute kidney injury with disease severity and mortality in COVID-19: a systematic review and meta-analysis. Cureus.

[6] Menon T, Sharma R, Earthineni G (2021). Association of gastrointestinal system with severity and mortality of COVID-19: a systematic review and meta-analysis. Cureus.

[7] Kataria S, Sharif A, Ur Rehman A, Ahmed Z, Hanan A (2020). COVID-19 induced acute pancreatitis: a case report and literature review. Cureus.

[8] Shah K, Mann S, Singh R, Bangar R, Kulkarni R (2020). Impact of COVID-19 on the mental health of children and adolescents. Cureus.

[9] Menon T, Gandhi SA, Tariq W (2021). Impact of chronic kidney disease on severity and mortality in COVID-19 patients: a systematic review and meta-analysis. Cureus.

[10] Bansal V, Mahapure KS, Mehra I (2021). Mortality benefit of convalescent plasma in COVID-19: a systematic review and meta-analysis. Front Med (Lausanne).

[11] Singh R, Shaik L, Mehra I, Kashyap R, Surani S (2020). Novel and controversial therapies in COVID-19. Open Respir Med J.

[12] Bansal V, Mahapure KS, Bhurwal A (2020). Mortality benefit of remdesivir in COVID-19: a systematic review and meta-analysis. Front Med (Lausanne).

[13] Neupane K, Ahmed Z, Pervez H, Ashraf R, Majeed A (2020). Potential treatment options for COVID-19: a comprehensive review of global pharmacological development efforts. Cureus.

[14] Khan I, Ahmed Z, Sarwar A, Jamil A, Anwer F (2020). The potential vaccine component for COVID-19: a comprehensive review of global vaccine development efforts. Cureus.

[15] Pinna P, Grewal P, Hall JP (2020). Neurological manifestations and COVID-19: experiences from a tertiary care center at the Frontline. J Neurol Sci.

[16] Singh R, Shiza ST, Saadat R, Dawe M, Rehman U (2021). Association of Guillain-Barre syndrome with COVID-19: a case report and literature review. Cureus.

[17] Radmard S, Epstein SE, Roeder HJ (2020). Inpatient neurology consultations during the onset of the SARS-CoV-2 New York City pandemic: a single center case series. Front Neurol.

[18] Mao L, Jin H, Wang M (2020). Neurologic manifestations of hospitalized patients with coronavirus disease 2019 in Wuhan, China. JAMA Neurol.

[19] (2022). Review Manager Web (RevMan Web). https://revman.cochrane.org/.

[20] Anand P, Al-Faraj A, Sader E (2020). Seizure as the presenting symptom of COVID-19: a retrospective case series. Epilepsy Behav.

[21] Canham LJ, Staniaszek LE, Mortimer AM, Nouri LF, Kane NM (2020). Electroencephalographic (EEG) features of encephalopathy in the setting of Covid-19: a case series. Clin Neurophysiol Pract.

[22] Chen W, Toprani S, Werbaneth K, Falco-Walter J (2020). Status epilepticus and other EEG findings in patients with COVID-19: a case series. Seizure.

[23] Delorme C, Paccoud O, Kas A (2020). COVID-19-related encephalopathy: a case series with brain FDG-positron-emission tomography/computed tomography findings. Eur J Neurol.

[24] Galanopoulou AS, Ferastraoaru V, Correa DJ (2020). EEG findings in acutely ill patients investigated for SARS-CoV-2/COVID-19: a small case series preliminary report. Epilepsia Open.

[25] Jain R, Young M, Dogra S (2020). COVID-19 related neuroimaging findings: a signal of thromboembolic complications and a strong prognostic marker of poor patient outcome. J Neurol Sci.

[26] Louis S, Dhawan A, Newey C, Nair D, Jehi L, Hantus S, Punia V (2020). Continuous electroencephalography characteristics and acute symptomatic seizures in COVID-19 patients. Clin Neurophysiol.

[27] Mahammedi A, Saba L, Vagal A (2020). Imaging of neurologic disease in hospitalized patients with COVID-19: an Italian multicenter retrospective observational study. Radiology.

[28] Petrescu AM, Taussig D, Bouilleret V (2020). Electroencephalogram (EEG) in COVID-19: a systematic retrospective study. Neurophysiol Clin.

[29] Pellinen J, Carroll E, Friedman D (2020). Continuous EEG findings in patients with COVID-19 infection admitted to a New York academic hospital system. Epilepsia.

[30] Pilato MS, Urban A, Alkawadri R (2022). EEG findings in coronavirus disease. J Clin Neurophysiol.

[31] Romero-Sánchez CM, Díaz-Maroto I, Fernández-Díaz E (2020). Neurologic manifestations in hospitalized patients with COVID-19: the ALBACOVID registry. Neurology.

[32] Kremer S, Lersy F, de Sèze J (2020). Brain MRI findings in severe COVID-19: a retrospective observational study. Radiology.

[33] Tomlins J, Hamilton F, Gunning S, Sheehy C, Moran E, MacGowan A (2020). Clinical features of 95 sequential hospitalised patients with novel coronavirus 2019 disease (COVID-19), the first UK cohort. J Infect.

[34] Li Y, Li H, Fan R (2016). Coronavirus infections in the central nervous system and respiratory tract show distinct features in hospitalized children. Intervirology.

[35] Keshavarzi A, Janbabaei G, Kheyrati L, Ghavamabad LH, Asadi-Pooya AA (2021). Seizure is a rare presenting manifestation of COVID-19. Seizure.

[36] Santos de Lima F, Issa N, Seibert K (2021). Epileptiform activity and seizures in patients with COVID-19. J Neurol Neurosurg Psychiatry.

[37] Hung EC, Chim SS, Chan PK (2003). Detection of SARS coronavirus RNA in the cerebrospinal fluid of a patient with severe acute respiratory syndrome. Clin Chem.

[38] Saad M, Omrani AS, Baig K (2014). Clinical aspects and outcomes of 70 patients with Middle East respiratory syndrome coronavirus infection: a single-center experience in Saudi Arabia. Int J Infect Dis.

[39] Favas TT, Dev P, Chaurasia RN (2020). Neurological manifestations of COVID-19: a systematic review and meta-analysis of proportions. Neurol Sci.

[40] Asadi-Pooya AA, Simani L, Shahisavandi M, Barzegar Z (2021). COVID-19, de novo seizures, and epilepsy: a systematic review. Neurol Sci.

[41] Cabezudo-García P, Ciano-Petersen NL, Mena-Vázquez N, Pons-Pons G, Castro-Sánchez MV, Serrano-Castro PJ (2020). Incidence and case fatality rate of COVID-19 in patients with active epilepsy. Neurology.

[42] Coperchini F, Chiovato L, Croce L, Magri F, Rotondi M (2020). The cytokine storm in COVID-19: an overview of the involvement of the chemokine/chemokine-receptor system. Cytokine Growth Factor Rev.

[43] Sureka RK, Gaur V, Gupta M (2021). Impact of COVID-19 on people suffering with epilepsy. Ann Indian Acad Neurol.

